# Collet–Sicard syndrome due to cervical artery dissection disclosed by high‐resolution magnetic resonance imaging

**DOI:** 10.1111/ene.16398

**Published:** 2024-07-19

**Authors:** Aikaterini Theodorou, Stefanos Lachanis, Georgia Papagiannopoulou, Maria Maili, Ioanna Pachi, Georgios Velonakis, Eleni Bakola, Sofia Vassilopoulou, Georgios Tsivgoulis

**Affiliations:** ^1^ Second Department of Neurology “Attikon” University Hospital, School of Medicine, National and Kapodistrian University of Athens Athens Greece; ^2^ Iatropolis Magnetic Resonance Diagnostic Centre Athens Greece; ^3^ Second Department of Radiology “Attikon” University Hospital, School of Medicine, National and Kapodistrian University of Athens Athens Greece; ^4^ First Department of Neurology “Eginition” University Hospital, School of Medicine, National and Kapodistrian University of Athens Athens Greece; ^5^ Department of Neurology University of Tennessee Health Science Center Memphis Tennessee USA

**Keywords:** cervical artery dissection, Collet–Sicard syndrome, high‐resolution MRI, lower cranial nerve palsies

## Abstract

**Background and purpose:**

Cervical artery dissection (CAD) represents a leading cause of unilateral lower cranial nerve IX–XII palsy, known as Collet–Sicard syndrome (CSS). High‐resolution magnetic resonance imaging (HR‐MRI) is widely used in the evaluation of patients with CAD, providing information regarding vessel wall abnormalities and intraluminal thrombus.

**Methods:**

We present a patient with palsy of multiple lower cranial nerves in the context of CSS, attributed to unilateral spontaneous internal carotid artery dissection.

**Results:**

We describe a 68‐year‐old man with unremarkable previous history, who presented with subacute, gradually worsening dysphagia and hoarse voice. Clinical examination revealed right‐sided palsy of cranial nerves IX–XII. Three‐dimensional fat‐saturated black‐blood T1‐weighted high‐resolution vessel wall imaging disclosed spontaneous dissection with intramural hematoma along the distal right internal carotid artery. Neck MRI showed inward displacement of right aryepiglottic fold, right pyriform sinus dilatation, and right true vocal cord in middle position, indicative of right vagus nerve palsy, atrophy of right trapezius and sternocleidomastoid muscles, due to right spinal accessory nerve palsy, and unilateral tongue atrophy with fatty infiltration, characteristic for right hypoglossal nerve palsy.

**Conclusions:**

This case highlights the utility of high‐resolution vessel wall imaging and especially fat‐saturated T1‐weighted black‐blood SPACE (sampling perfection with application‐optimized contrast using different flip‐angle evolutions) sequences in the accurate diagnosis of CAD, revealing the characteristic mural hematoma and intimal flap. HR‐MRI is also valuable in the recognition of indirect signs of lower cranial nerve compression.

## INTRODUCTION

Collet–Sicard syndrome (CSS) is an uncommon diagnosis, characterized by unilateral paralysis of the IX, X, XI, and XII cranial nerves [1]. Leading underlying etiology of this syndrome is cervical artery dissection (CAD) [1]. High‐resolution magnetic resonance imaging (HR‐MRI) represents a very promising and versatile technique, contributing to the accurate and noninvasive detection of the characteristic intramural hematoma and intimal flap and the identification of indirect signs of lower cranial nerve compression [2].

## METHODS

We report the case of a 68‐year‐old man with CSS and characteristic cranial nerve IX–XII palsy. Unilateral spontaneous internal carotid artery (ICA) dissection as the underlying causative mechanism was disclosed using HR‐MRI.

## CASE DESCRIPTION

A 68‐year‐old man presented to the emergency department of our hospital due to 1‐month history of gradually worsening swallowing compromise for both liquids and solids and hoarse voice. Right‐sided headache and neck pain were also present on symptom onset. Blood pressure levels were 135/75 mmHg, and heart rate was 65 bpm. The patient was engaged in agricultural work, but his previous medical history was unremarkable. No trauma or chiropractic manipulations were reported. He denied tobacco, alcohol, or drug use, and he had no history of systematic medication intake. Neurological examination confirmed the dysphagia and also revealed whispering/guttural speech and absent gag reflex indicative of glossopharyngeal (IX) and vagus (X) nerve palsy. Moreover, the patient had mild right shoulder drop, suggesting right accessory (XI) nerve impairment. Right‐sided atrophy of the tongue without fasciculations and deviation in the contralateral side were indicative of hypoglossal (XII) cranial nerve palsy. Symptoms such as ptosis or pupil constriction were not observed. Electronic fiberoptic laryngoscopy revealed right vocal cord paralysis as well.

**FIGURE 1:**
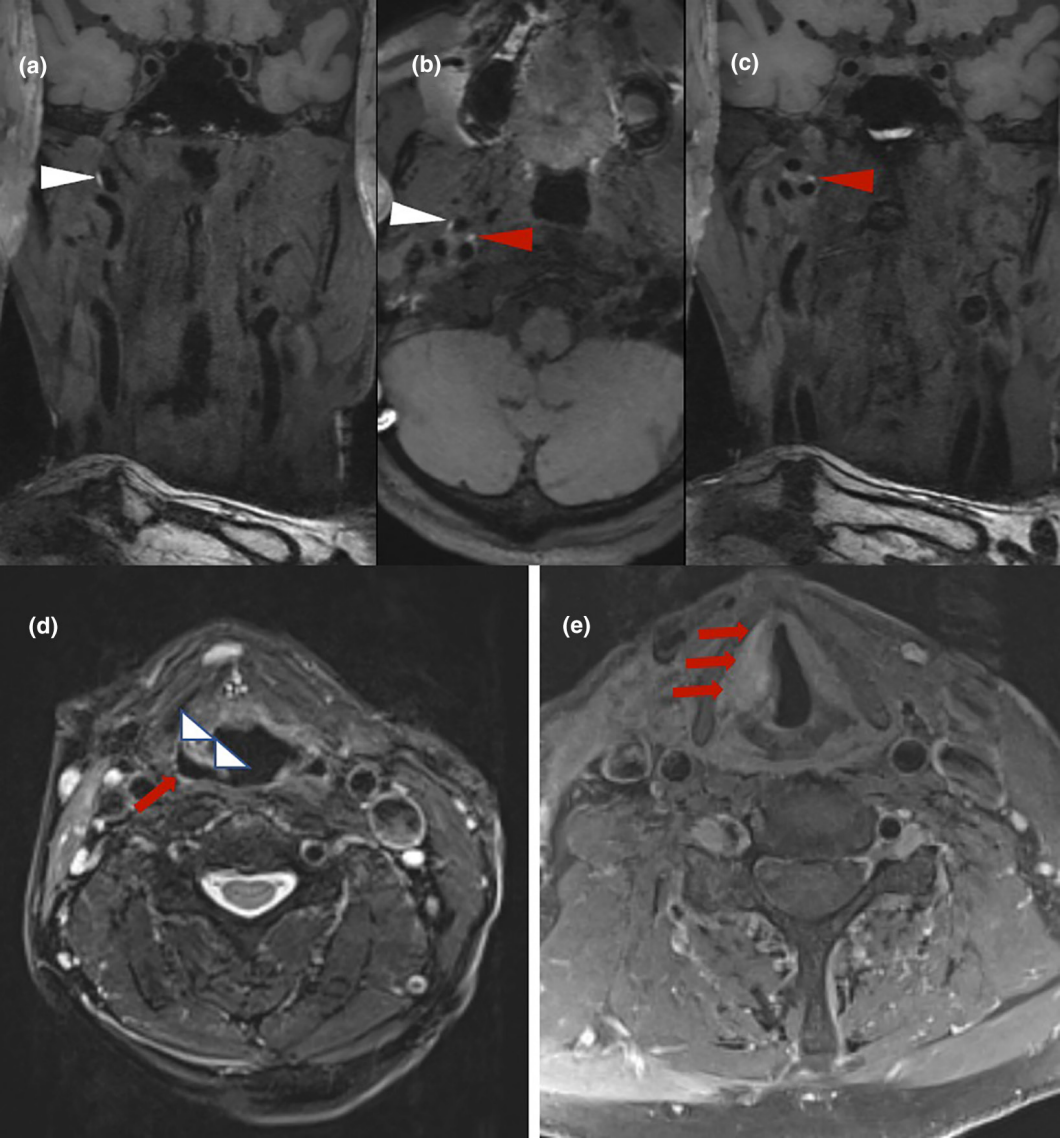
Neuroimaging findings disclosing right internal carotid artery dissection and right vagus (X) nerve palsy. Fat‐saturated black‐blood T1‐weighted high‐resolution (3 T) vessel wall imaging with multiplanar reconstruction shows right internal carotid artery dissection with intramural hematoma (a–c). T2‐weighted images reveal inward displacement of aryepiglottic fold (d; white arrowheads), pyriform sinus dilatation (d; red arrow), and right vocal cord in middle position (e), indicating right vagus nerve palsy.

**FIGURE 2:**
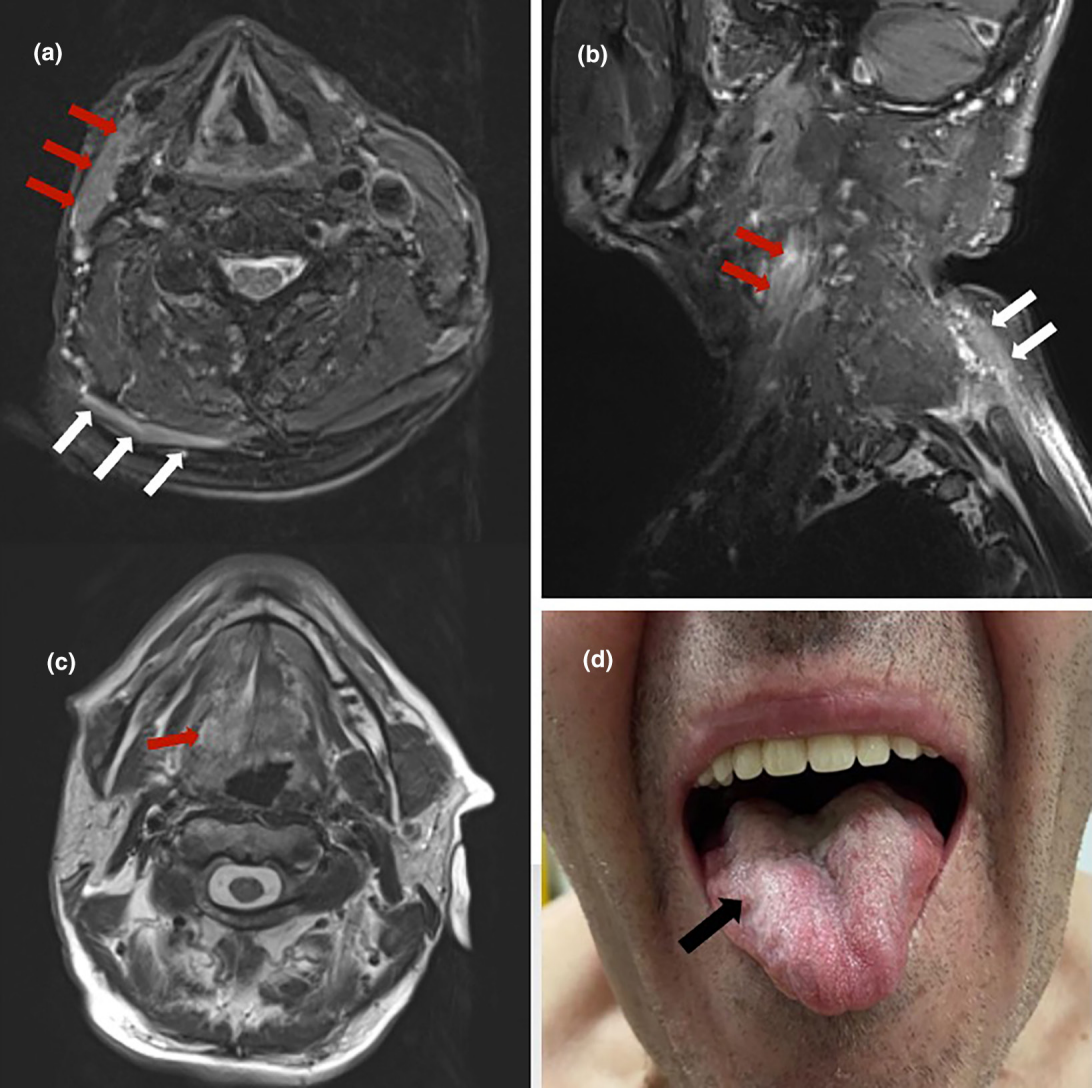
Neuroimaging findings and clinical manifestations indicating right spinal accessory nerve (XI) and hypoglossal (XII) nerve palsies. T2‐weighted and T1‐space images show right trapezius and sternocleidomastoid muscle atrophy (a, b; red arrows for sternocleidomastoid muscle and white arrows for trapezius muscle), findings compatible with spinal accessory nerve palsy. T2‐weighted magnetic resonance images (c) and neurological examination (d) revealed right‐sided atrophy of the tongue, indicating right hypoglossal nerve palsy.

The patient underwent brain MRI, which was negative for ischaemic and haemorrhagic lesions, and carotid ultrasound was also performed, documenting unremarkable findings. Three‐dimensional fat‐saturated black‐blood T1‐weighted high‐resolution (3 T) vessel wall MRI disclosed spontaneous dissection with intramural hematoma along the distal right ICA (Figure 1a–c). T2‐weighted sequences revealed (i) inward displacement of right aryepiglottic fold, right pyriform sinus dilatation, and right true vocal cord in middle position (Figure 1d,e), indicative of right vagus nerve palsy; (ii) atrophy of right trapezius and sternocleidomastoid muscles, due to right spinal accessory nerve palsy (Figure 2a,b); and (iii) unilateral tongue atrophy with fatty infiltration, characteristic for right hypoglossal nerve palsy (Figure 2c,d).

CSS diagnosis with the characteristic unilateral lesion of cranial nerves IX–XII was made and attributed to spontaneous CAD. Digital subtraction angiography (DSA) was not performed, because the patient refused consent. The patient was started on antiplatelet therapy (acetylsalicylic acid 100 mg/day) and was discharged home. Gradual symptomatic resolution was observed in the following 8 months, including improvement in speech and swallowing.

## DISCUSSION

CSS is a rare syndrome, first described in 1915 by Frederic Collet, an otologist, and few years later by Jean Sicard, a neurologist [3, 4]. They reported soldiers with this syndrome, who had been shot during World War I.

CSS is the clinical manifestation of lesions affecting the jugular foramen, parapharyngeal retrostyloid space, and hypoglossal canal with characteristic sparing of the sympathetic chain. Unilateral lesions affecting IX–XII cranial nerves in combination with ipsilateral Horner syndrome are called Villaret syndrome [5]. Carotid injuries causing CSS are mainly localized more distally compared to those causing Villaret syndrome, and near to the jugular foramen. The vast majority of the cervical dissections are subintimal. However, lower cranial nerve palsies due to CADs have been mostly associated with subadventitial wall hematomas, originating from a rupture from the vasa vasorum and leading to expansive vessel pathologies [6]. CSS has been attributed to multiple underlying etiologies, including head injuries (occipital fracture or burst fracture of the first cervical vertebra) and metastases at the base of the skull due to prostate cancer, breast carcinoma, or multiple myeloma [5]. Vascular etiologies, including spontaneous or traumatic ICA dissection, with or without ICA pseudoaneurysm or dissecting aneurysm, fibromuscular dysplasia, and thrombosis of the internal jugular vein have also been commonly implicated in this syndrome [5, 7]. Uncommon causes of the syndrome represent the iatrogenic complications and inflammatory processes, such as polyarteritis nodosa [5].

Headache and ipsilateral shoulder and neck pain could be the first CSS clinical manifestations, commonly attributable to traumatic or vascular etiologies [7]. Other manifesting symptoms include dysphagia, impaired gustation, reduced sensation over the posterior third of tongue, and absent gag reflex, as well as ipsilateral paralysis and anesthesia of the larynx, resulting in dysphonia and hoarseness due to glossopharyngeal (IX) and vagus (X) nerve palsy [7, 8]. Accessory (XI) nerve paralysis mainly results in sternocleidomastoid and trapezius muscle weakness and atrophy in the subacute phase, associated with affecting rotation of the neck and head and characteristic shoulder drop [7, 8]. Moreover, hypoglossal (XII) nerve palsy may manifest with tongue deviation to the side of the nerve paralysis and ipsilateral tongue atrophy, even with fasciculations in later stages [7, 8]. Major complications in these situations include weight loss and pulmonary infections due to prominent dysphagia.

Although ultrasonography can be used in the diagnosis of CADs, this should be confirmed with magnetic resonance angiography. Moreover, it is worth noting that routine carotid ultrasound could likely miss a dissection of the distal ICA behind the mandible, leading to misdiagnosis. On the other hand, computed tomographic angiography represents a very widely and rapidly available diagnostic method, which however could not add additional information in patients who have already undergone HR‐MRI. Despite some disadvantages, including invasiveness, high cost, and risk of stroke, DSA represents an alternative reliable imaging modality for the diagnosis of extracranial dissections [7]. DSA is mainly performed for investigation of the intraluminal thrombus and to grade stenosis, for evaluation of the collateralization in total occlusions and probable endovascular treatment [7]. However, it has been currently replaced by noninvasive and widely available HR‐MRI vessel wall imaging. Fat‐saturated T1‐weighted black‐blood SPACE (sampling perfection with application‐optimized contrast using different flip‐angle evolutions) sequences can easily diagnose different characteristics of a cervical dissection, including intramural hematoma, luminal flap, false lumen, long tapered stenosis, and dissecting aneurysm, providing valuable, clinically relevant information [9].

Moreover, the contribution of HR‐MRI in the recognition of imaging abnormalities as a consequence of muscular denervation secondary to V, VII, X, and XI motor cranial denervation should be acknowledged [10]. Radiological findings, including medial positioning of the ipsilateral aryepiglottic fold, dilatation of the ipsilateral pyriform sinus, anteromedial positioning of the ipsilateral arytenoid cartilage, and paramedian positioning of the ipsilateral vocal cord, represent some of the most commonly detected MRI findings among patients with vagus nerve palsy [10]. In cases of accessory nerve palsy, characteristic neuroimaging findings can be detected in the later stages, where atrophy of the trapezius and sternocleidomastoid muscles are present, with associated compensatory hypertrophy of the levator scapulae muscle combined in subacute phase with slight gadolinium enhancement [10]. Temporal associated MRI findings have been described in cases of hypoglossal nerve palsy. In early stages “oedemalike” changes and fatty atrophy of the tongue in T1‐weighted sequences have been described. T2‐weighted hyperintensity with associated enhancement are detected in the subacute or later phase, sometimes misleading to inflammatory, traumatic, or even neoplastic etiologic investigation of the tongue atrophy [10].

The treatment of CSS is etiological. In the case of coexistent minor ischemic strokes or patients with transient ischemic attacks, international guidelines on management of extracranial artery dissections recommend a course of dual antiplatelet therapy with aspirin and clopidogrel, restricted to a few weeks and followed by single antiplatelet treatment [11]. In postacute phase, use of endovascular/surgical treatment in patients with recurrent ischemic events (despite optimal conservative treatment) and in patients with expanding aneurysms causing worsening of muscle/nerve compression may be individualized [12].

In summary, this case highlights the utility of HR‐MRI in investigation of CSS provoked by underlying extracranial spontaneous carotid artery dissection, based on characteristic neuroimaging findings [13]. This imaging modality may simultaneously document the diagnosis of dissection by detecting intramural hematoma and also identifying indirect signs of lower cranial nerve compression.

## AUTHOR CONTRIBUTIONS

**Aikaterini Theodorou:** Writing – original draft; data curation; methodology; conceptualization. **Stefanos Lachanis:** Writing – original draft; data curation. **Georgia Papagiannopoulou:** Writing – original draft; data curation. **Maria Maili:** Writing – review and editing. **Ioanna Pachi:** Writing – review and editing. **Georgios Velonakis:** Writing – review and editing. **Eleni Bakola:** Writing – review and editing. **Sofia Vassilopoulou:** Writing – review and editing. **Georgios Tsivgoulis:** Supervision; conceptualization; writing – original draft; methodology.

## CONFLICT OF INTEREST STATEMENT

All authors report no disclosures.

## ETHICS STATEMENT

Ethical approval from our institutional ethics committee is not required for case reports.

## INFORMED CONSENT

The patient has provided written informed consent for the publication of this case report.

## Data Availability

The data that support the findings of this study are available on request from the corresponding author.
